# Overnight Orthokeratology: Technology, Efficiency, Safety, and Myopia Control

**DOI:** 10.1155/2019/2607429

**Published:** 2019-04-08

**Authors:** César Villa-Collar, Gonzalo Carracedo, Zhi Chen, José M. Gonzalez-Méijome

**Affiliations:** ^1^Department of Pharmacy, Biotechnology, Nutrition, Optics and Optometry, Faculty of Biomedical and Health Sciences, Universidad Europea de Madrid, Madrid, Spain; ^2^Department of Optics II (Optometry and Vision), Faculty of Optics and Optometry, Universidad Complutense de Madrid, Madrid, Spain; ^3^Department of Ophthalmology and Vision Science, Eye and ENT Hospital, Fudan University, Shanghai, China; ^4^Clinical & Experimental Optometry Research Lab (CEORLab), Center of Physics, Universidade do Minho, Braga, Portugal

Modern orthokeratology differs significantly from the original technique back in the 1960s. Over the last 3 decades, new materials, lens designs, manufacturing processes, fitting techniques, and instruments for the analysis of corneal changes have been developed and have contributed to its evolution. Nowadays, orthokeratology is carried out using the contact lenses during sleep hours (overnight orthokeratology (OOK)), and it is approved by FDA for the treatment of myopia, up to 6 dioptres. There are reports of some designs that allow treatment of myopia up to 10–12 dioptres and hyperopia up to 3 dioptres, and recent toric designs, either in the optical zone or in the periphery of the lens, allow correction of astigmatism above 1.75 dioptres up to 3.50 dioptres though those treatments are performed off-label. Currently, even some cases of presbyopia may be solved with the help of OOK [1, 2]. However, the greatest impact of this technique in recent years is its application as a method for the control of myopia progression, either on its own or in combination with low-dose atropine [3, 4]. Recently, a published report written by the American Academy of Ophthalmology concludes that orthokeratology is effective for myopia control and potentially has a greater effect when it is applied in patients aged 6 to 8 years [5].

Moreover, orthokeratology lenses represent 1.2% of all contact lenses fitting operations, although there are significant differences from one country to another, from an almost complete lack of lens fitting in countries like Brazil, Egypt, or Indonesia, up to 6% in the Netherlands. OOK lenses are fitted to younger patients (25.00 ± 12.8 years), as opposed to non-OOK lenses (39.8 ± 14.9 years) [6].

The period from 2013 to 2017 has witnessed a strong increase in the number of published papers regarding this technique, where almost 40% of all the papers about orthokeratology were published over this time period [2]. This is the reason why we suggested *Journal of Ophthalmology* to dedicate a special issue to this contact lens fitting technique.

This special issue contains 6 papers focusing on different aspects, among them, quality of vision, corneal changes according to lens geometry or the involvement of eye surface during their use.

X. Wang et al. consider that OOK influences tear meniscus height and tear break-out time. However, in their opinion, the operation of Meibomian glands is not affected. On the other hand, Z. Chen et al. warn about the increase in corneal toricity after discontinuing the treatment, which is associated with an increase in refractive astigmatism. H.-C. Guo et al. conclude that the OOK reduces the modulation transfer function (MTF) and the contrast sensitivity function (CSF) of low frequencies, as a result of the increase in intraocular scattering as well as higher-order aberrations. Furthermore, CSF values at higher spatial frequencies suffer significant fluctuations one day after the OOK contact lenses have been used for a whole month. J. Jiang et al. conclude that the design of toric lenses may decrease the size of the off-centring in patients with moderate to high corneal astigmatism. M. A. Sánchez-Tena et al. show the status of the orthokeratology information flow by analysing the so-called citation networks. Finally, G. Carracedo et al. verified that a diameter of 5 mm of the optical area of OOK lenses entails a smaller area of treatment and a greater and more powerful midperipheral ring; in turn, this increases the fourth-order spherical aberration which only affects the CSF, without differences as to visual acuity and subjective vision, when compared with a larger optical zone exceeding 6 mm.
